# Vitamin K2, a Naturally Occurring Menaquinone, Exerts Therapeutic Effects on Both Hormone-Dependent and Hormone-Independent Prostate Cancer Cells

**DOI:** 10.1155/2013/287358

**Published:** 2013-08-24

**Authors:** Abhilash Samykutty, Aditya V. Shetty, Gajalakshmi Dakshinamoorthy, Ramaswamy Kalyanasundaram, Gouxing Zheng, Aoshuang Chen, Maarten C. Bosland, André Kajdacsy-Balla, Munirathinam Gnanasekar

**Affiliations:** ^1^Department of Biomedical Sciences, University of Illinois, Rockford, IL 61107, USA; ^2^Department of Pathology, University of Illinois at Chicago, Chicago, IL 60612, USA

## Abstract

In recent years, several studies have shown that vitamin k2 (VK2) has anticancer activity in a variety of cancer cells. The antitumor effects of VK2 in prostate cancer are currently not known. In the present study, we sought to characterize the anticancer potential of VK2 in both androgen-dependent and -independent prostate cancer cells. Our investigations show that VK2 is able to suppress viability of androgen-dependent and androgen-independent prostate cancer cells via caspase-3 and -8 dependent apoptosis. We also show that VK2 treatment reduces androgen receptor expression and PSA secretion in androgen-dependent prostate cancer cells. Our results also implicate VK2 as a potential anti-inflammatory agent, as several inflammatory genes are downregulated in prostate cancer cells following treatment with VK2. Additionally, AKT and NF-kB levels in prostate cancer cells are reduced significantly when treated with VK2. These findings correlated with the results of the Boyden chamber and angiogenesis assay, as VK2 treatment reduced cell migration and angiogenesis potential of prostate cancer cells. Finally, in a nude mice model, VK2 administration resulted in significant inhibition of both androgen-dependent and androgen-independent tumor growth. Overall, our results suggest that VK2 may be a potential therapeutic agent in the treatment of prostate cancer.

## 1. Introduction

Prostate cancer is the most common solid malignancy in men. In the USA, it is estimated that 241,740 new cases and 28,170 deaths will occur in 2012 [1]. Prostate cancer is currently treated with a combination of surgery, androgen ablation or radiation therapy. Those undergoing hormonal therapy eventually develop aggressive hormone unresponsive disease. Hence, one of the major focuses in prostate cancer research is the discovery of better chemotherapeutic agents for the advanced hormone-resistant, metastatic form of this disease.

Vitamin k is a fat soluble vitamin that plays a major role in the clotting cascade by acting as a coenzyme for a vitamin k dependent carboxylase that catalyzes the carboxylation of glutamic acid residues to produce gamma-carboxyglutamic acid [2]. Vitamin k also appears to work in regulation of bone metabolism through a similar mechanism via gamma carboxylation of bone matrix proteins [3]. There are two naturally-occuring vitamin k compounds, vitamin k1 (phylloquinone) and vitamin k2 (menaquinone). Interestingly, vitamin k2 (VK2) intake seems to be associated with greater benefits of reduced coronary calcification when compared to vitamin k1 consumption [4]. In recent years, various reports have shown that VK2 has antioncogenic effects in various cancer cell lines, including leukemia, lung cancer, ovarian cancer, and hepatocellular cancer [5–9]. Although the exact mechanisms by which VK2 exert its antitumor effect are still unclear, processes such as cell cycle arrest, apoptosis and induction of differentiation appear to contribute to the therapeutic effects of VK2 [5–9]. The antitumor effects of VK2 have been most extensively studied in hepatocellular cancer. Yamamoto and colleagues showed that downregulation of hepatoma-derived growth factor is partially responsible for the growth suppression properties of VK2 in hepatocellular cell lines [10]. In another study, Otsuka and colleagues showed that VK2 inhibits growth and invasion of hepatocellular cell lines via activation of protein kinase A [11]. Recent studies also suggest a role for VK2 in the prevention of cancer, as a randomized trial of 43 women with viral hepatitis treated with high dose VK2 showed an 80% decreased risk of developing hepatocellular carcinoma [12].

In view of VK2 potential to reduce osteoporosis [13] and atherosclerosis risk [4] and given the fact that these two pathologies are frequently associated with prostate cancer patients undergoing hormonal therapy [14, 15], development of VK2 as a treatment strategy for prostate cancer would have far reaching impact on prostate cancer patients. Previously, Nimptsch et al. showed an inverse relationship between dietary intake of VK2 and risk of prostate cancer [16]. Interestingly, serum undercarboxylated osteocalcin (ucOC), a biomarker of vitamin k status, is inversely associated with VK2 intake and the development of advanced prostate cancer [17]. These studies thus suggest that the intake of VK2 may be beneficial in preventing the progression of prostate cancer. Moreover, VK2 is also shown to enhance the chemotherapeutic efficacy of conventional anticancer drug Sorafenib in hepatocellular carcinoma [18]. Unlike its synthetic counterpart, vitamin k3, there are no known side effects associated with ingestion of high doses of VK2 [19]. To date, however, no studies have been conducted to assess the therapeutic potential of VK2 in the treatment of prostate cancer. To our knowledge, this is the first comprehensive study which demonstrates the therapeutic potential of VK2 against both forms of prostate cancer (hormone dependent and hormone independent) using *in vitro* and *in vivo* models with mechanistic details of VK2 action. 

## 2. Materials and Methods

### 2.1. Ethics Statement

Animal experiments were performed in this study according to the guidelines set for the care and use of laboratory animals and with the rules formulated under the Animal Welfare Act by the United States Department of Agriculture (USDA) and by adopting ARRIVE guidelines [20]. The protocol was approved by the IACUC Committee of the University of Illinois, College of Medicine at Rockford, and animal studies performed at a facility accredited by AAALAC and USDA.

### 2.2. Chemicals and Reagents

Fetal calf serum (FCS), RPMI-1640, and minimum essential medium (MEM) were obtained from American Type Cell Culture (ATCC), Manassas, VA. Keratinocyte serum-free medium (KSFM) was obtained from Life Technologies, Grand Island, NY. Vitamin K2 (VK2) was purchased from Sigma-Aldrich (St. Louis, MO). Cell viability assay kit was acquired from Dojindo Molecular Technologies Inc., Gaithersburg, MD. Annexin V-FITC apoptosis detection Kit was obtained from MBL international, Woburn, MA.

### 2.3. Cell Lines and Cell Culture

RWPE-1, LNCaP, DU145, and 22RV1 cell lines were obtained from ATCC. LNCaP, DU145, and 22RV1 cells were cultured in RPMI medium supplemented with 10% FCS and 50 *μ*g/mL gentamycin. RWPE-1 cells were cultured in KSFM supplemented with bovine pituitary extract and EGF. For all experiments, 1 × 10^5^ cells/mL were seeded and grown for 48 h before experimental treatments. Cells were maintained at 37°C, 5% CO_2_ environment. 

### 2.4. Cell Viability Assay

RWPE-1, LNCaP, DU145, and 22RV1 cells were seeded in 96-well tissue culture plates and incubated until cells attached to wells. All the cells were then treated and incubated with different concentrations of VK2 (1–500 *μ*M) for 2, 4, and 8 days. Cell viabilities were determined using a cell counting kit-8 (CCK-8) from Dojindo Molecular Technologies. 10 *μ*L CCK-8 solution was added to the VK2 treated plates and incubated for 3 h. Optical density was measured at 450 nm using a BIO-RAD microplate reader model 680.

### 2.5. PSA Assay

PSA was performed using the supernatants collected from LNCaP cells treated with varying concentrations of VK2 for 48 h. Prostate specific antigen secretion (ng/mL) was determined using a Human Prostate Specific Antigen ELISA kit purchased from Abnova. 

### 2.6. Caspase Activation Assay

LNCaP, 22RV1, and DU145 cells were seeded in 96-well tissue culture plates and incubated until they reached 50% confluency. Plates were then treated and incubated with 100 *μ*M VK2 for 2 days. Each plate was then incubated with 2 *μ*L fluorescently-labeled caspase probe (NIR-FLIVO 747 *In Vivo* Apoptosis Tracer, Immunochemistry Technologies, LLC, Bloomington, MN) for 15 minutes. Cells were washed with 100 *μ*L 1X PBS to remove caspase substrate. Following this, 100 *μ*L 1xPBS was added to the wells. Plates were read using a LI-COR Odyssey machine V3.0 to detect global caspase activation.

### 2.7. Caspase Activity

Caspase-3 and Caspase-8 activities were measured by using colorimetric assay kits (R&D) systems, Minneapolis, MN). Cells were washed with ice cold PBS and Caspase-3 and Caspase-8 activities were determined. Caspase colorimetric substrates DEVD-pNA (Caspase-3) or IETD-pNA (Caspase-8) were added to the cell lysate and assays were performed in a 100 *μ*L volume in 96-well flat bottomed plates. Chromophore-p-nitroanilide is released as a result of cleavage of substrates by caspase activity. The caspase enzymatic activity in the cell lysate is directly proportional to the chromophore formation, which was quantified spectrophotometrically at a wavelength of 405 nm using a microplate reader. Data were corrected for the background values that had no substrate or cell lysate added. 

### 2.8. Annexin V-FITC Staining for Apoptosis

LNCaP, 22RV1, and DU145 cells were cultured in an 8-chamber Culture Slide in RPMI-1640 medium supplemented with 10% fetal bovine serum (FBS) and gentamycin until cells attached to wells. Cells were then treated and incubated with 100 *μ*M of VK2 for 2 days. Apoptosis was determined using an Annexin V-FITC apoptosis detection kit from BioVision Inc. Cells were stained by adding 500 *μ*L of binding buffer, 5 *μ*L Annexin, and 5 *μ*L PI to each well and incubating in the dark at room temperature for 5–10 minutes. Binding buffer, Annexin, and PI were then removed from wells along with the chamber. 2-3 drops of 1xPBS was added to each section of the slide and covered. Slide was then analyzed using a fluorescence microscope.

### 2.9. Western Blot Analysis

LNCaP, 22RV1, and DU145 cells treated with varying concentrations of VK2 for 48 h. Cells were lysed with sample solubilizing buffer and subjected to SDS-PAGE, transferred to nitrocellulose membrane and probed with anti p65 (MBL International Inc), antiphospho-Akt (Cell signaling Technologies), antiAndrogen Receptor (Thermo Scientific), and anti *β*-Actin antibodies (Sigma Aldrich). Cells treated with 0.1% DMSO solvent served as controls. 

### 2.10. Real Time Quantification of Gene Expression

Quantification of gene expression of HMGB1, IL-6, IL-8, VEGF-A, AR, and RAGE was determined using real time PCR technique. For gene expression studies, LNCaP, DU145, and 22RV1 cells were incubated for 48 h in presence of VK2 at various concentrations. Following treatment, the cells were lysed with Trizol reagent and the RNA extracted. Untreated cells served as controls. From the RNA samples, cDNA was synthesized using SA Biosciences PCR kit. A SYBR green based gene specific real time PCR quantification kit (SA Biosciences) was used to analyze the expression of HMGB1, IL-6, IL-8, VEGF-A, AR, and RAGE genes. Real time amplification of genes was performed in Applied Biosystems 7300 Real time PCR instrument.

### 2.11. Boyden Chamber Assay

DU145 and 22RV1 cells were seeded in a Transwell (Corning) plate and treated with 50, 75 or 100 *μ*M of VK2 for 24 h. Cells were then removed from the top of the membrane using a pipette and any remaining cells were removed using a cotton swab (Q-tip). A HEMA 3 staining set from Thermo Fisher Scientific was used to fix and stain the migrated cells. Following this, each membrane was rinsed with water and any remaining stain was removed from the top of each membrane using a Q-tip. Membranes were analyzed for cell migration using a light microscope (Nikon).

### 2.12. *In Vivo* Matrigel Plug Assay

Six-week-old nude male mice weighing approximately 20 grams were maintained in the Animal Facility at the University of Illinois at Rockford College of Medicine. All experimental procedures using animals were approved by the Institutional Animal Care and Use Committee of the University of Illinois at Rockford College of Medicine. Nude mice were injected subcutaneously with 200 *μ*L of Matrigel along with 1 × 10^6^ 22RV1 or DU145 cells and either 50, 75, or 100 *μ*M of VK2 or the equivalent volume of water. Six mice were used in the treatment and control groups, respectively. Fourteen days later, mice were sacrificed by carbon dioxide inhalation followed by exsanguination, and matrigel plug was removed. To quantitate the formation of functional blood vessels, in the matrigel plug the amount of hemoglobin was measured using Drabkin hemoglobin assay as previously described [21].

### 2.13. Animals

Six-week-old nude male mice weighing approximately 20 grams were maintained in the Animal Facility at the University of Illinois at Rockford College of Medicine. All experimental procedures using animals were approved by the Institutional Animal Care and Use Committee of the University of Illinois at Rockford College of Medicine. LNCaP (5 × 10^6^), 22RV1 (1 × 10^6^) or DU145 (1 × 10^6^) cells in 0.1 mL of matrigel (Becton Dickinson) were injected subcutaneously into the flank regions of the nude mice and tumors were allowed to grow. To assess the effect of VK2 on tumor growth, VK2 or control treatments began once the tumor reached 50 mm^3^ in size. Because it is estimated that mice of this age drink approximately 30 mL of water per day, VK2 was added at 20 mg/kg/day to water bottles. Six mice were used in the treatment and control groups, respectively. Mice were treated for a total of 6 weeks. Following this, mice were sacrificed by carbon dioxide inhalation followed by exsanguination. Tumors were then excised and measured for mass and volume parameters. Tumor volumes were calculated by the formula: (Volume = 0.5 × (Width) 2 × length). This *in vivo* experiment was performed in concordance with ARRIVE guidelines [20]. 

### 2.14. Statistics

Data were compared using Graph Pad Prism 5 software. Data was compared using Student's “*t*”-test. *P* < 0.05 was considered statistically significant.

## 3. Results

### 3.1. VK2 Selectively Inhibits the Proliferation of LNCaP, 22RV1 and DU145 Prostate Cancer Cells *In Vitro*

**Figure 1:**
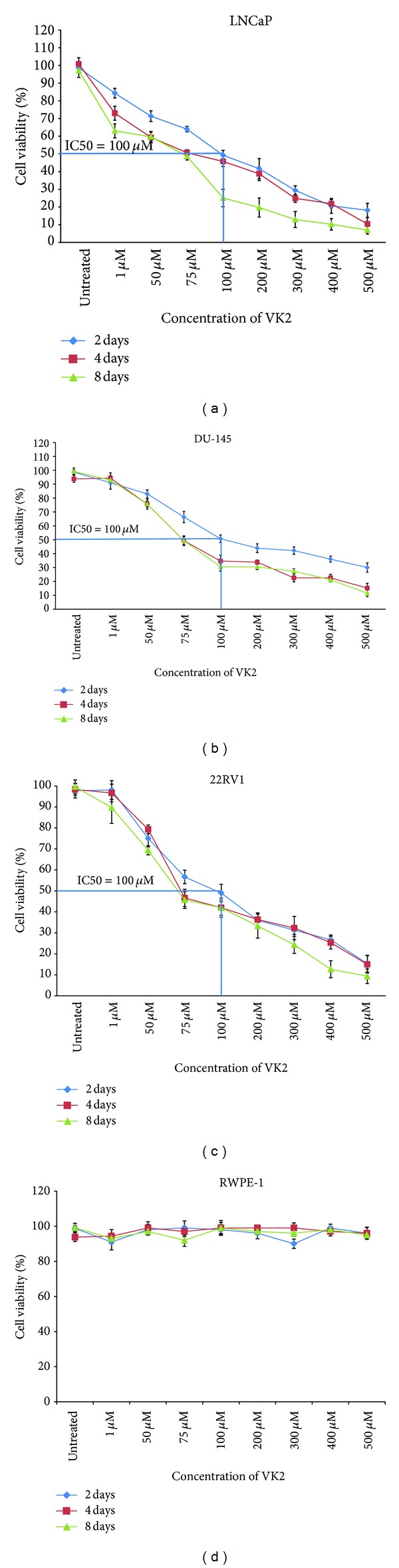
VK2 inhibits cell proliferation in androgen-dependent and androgen-independent prostate cancer cell lines. VK2 inhibits cell proliferation of LNCaP, 22RV1, and DU145 with an IC50 of about 100 *μ*M. Results showed that VK2 inhibited proliferation of both androgen-dependent (LNCaP) and androgen independent derived prostate cancer cells (22Rv1 and DU145) in a time- and dose-dependent manner. Nonmalignant prostate epithelial cells (RWPE-1) (Figure 1(d)) were not affected by VK2 treatment. Data presented here is representative of one of three similar experiments.

LNCaP, DU145 and 22RV1 cells were treated with varying concentrations of VK2 (1–500 *μ*M) at different time points (2, 4, and 8 days). Treatment in all these 3 prostate cancer cell lines resulted in a substantial reduction in viability or proliferation in a dose-dependent manner with an IC-50 of 100 *μ*M, as assessed by MTT (Figures 1(a), 1(b) and 1(c)). VK2 appears to selectively inhibit human prostate cancer cell lines as cell viability was not substantially reduced in normal prostate epithelial RWPE-1 cells (Figure 1(d)).

Based upon the results presented here, 100 *μ*M VK2 was used for further characterization studies with LNCAP, DU-145, and 22RV1 cells. Lower concentrations, 50 and 75 *μ*M VK2, were also used in further studies to help assess the underlying mechanisms by which VK2 inhibits cell proliferation.

### 3.2. VK2 Treatment Reduces Prostate Specific Antigen (PSA) Levels in Androgen-Dependent LNCaP Cells

**Figure 2:**
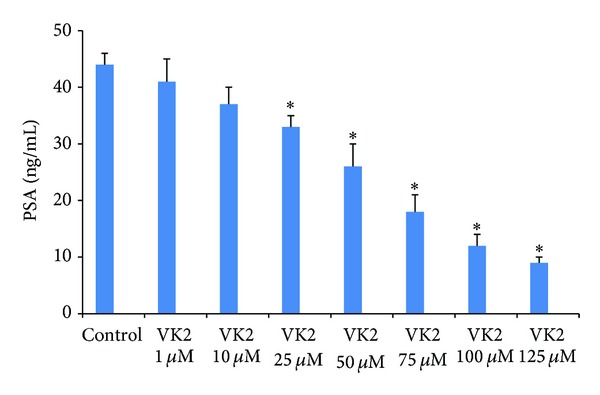
VK2 treatment down-regulates PSA expression in LNCaP cells at various concentrations. PSA assay results showed that VK2 has dose-dependent effects on the secretion of PSA (downstream target of AR) in LNCaP cells. *P < 0.05 compared to control LNCaP cells. Results shown here are representative of one of three similar experiments.

PSA is the gold standard marker used in diagnosis and monitoring treatment efficacy of prostate cancer. Our initial studies showed that AR positive LNCaP cells were sensitive to VK2 treatment. We, therefore, sought to determine the effects of VK2 on PSA levels in LNCaP cells. Our results show that VK2 treatment at concentrations from 50 to 100 *μ*M concentration significantly inhibited the secretion of PSA compared to untreated LNCaP cells (Figure 2). 

### 3.3. VK2 Induced Apoptosis in LNCaP, 22RV1, and DU145 Cells: Annexin-V Staining and Global Caspase Activation Assay

**Figure 3:**
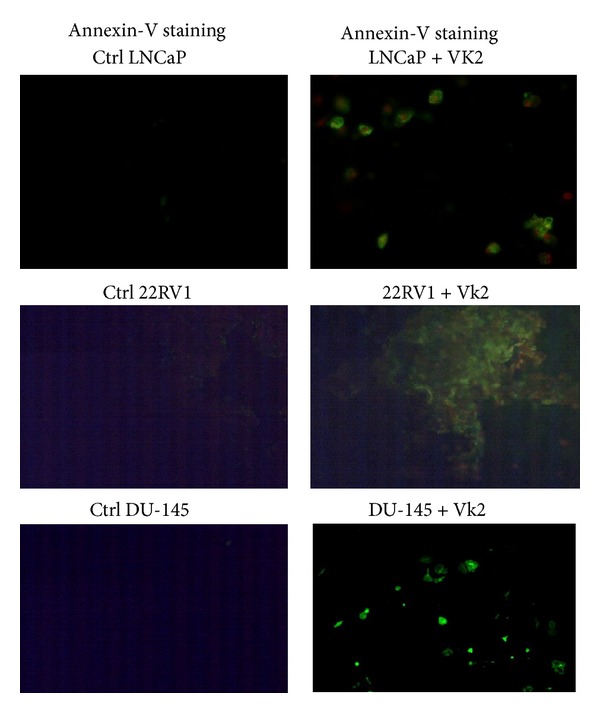
VK2 induces apoptosis in LNCaP, 22RV1 and DU145 cells. Annexin-V-FITC staining of the LNCaP, 22RV1, DU145 cells shows that cells treated with VK2 was positive for annexin binding as evident from fluorescence signal. Data presented here is representative of one of three similar experiments.

We then assessed if the reduction of cell viability in prostate cancer cell lines was due to increased apoptosis. LNCaP, 22RV1, and DU145 cells were treated with 100 *μ*M VK2 for 2 days and stained with Annexin V-FITC, and propidium iodide to visualize the cells under fluorescent microscope. Fluorescent microscopic analysis demonstrated that LNCaP, 22RV1, and DU-145 cells treated with VK2 were indeed undergoing apoptosis (Figure 3). 

**Figure 4:**
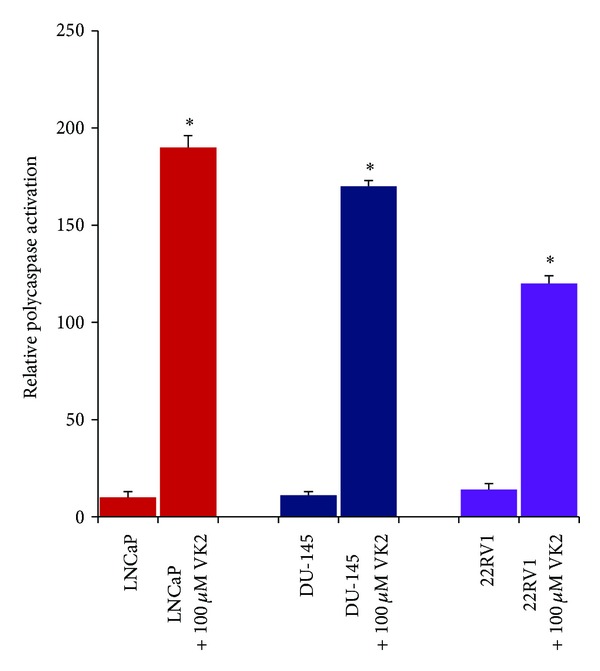
VK2 induced caspase activation in prostate cancer cell lines. A NIR-FLIVO 747 *In Vivo* Apoptosis Tracer, which is a cell-permeable fluorescent detector of active caspases, was employed to determine whether 100 *μ*M of VK2 activates caspase in prostate cancer cells. The LNCaP, 22RV1, and DU145 cell lines were treated with VK2 and then tested for caspase activation. Results showed that VK2 induced global caspase activation in LNCaP, 22RV1, and DU145 cells as early as 48 hours. *P < 0.05 compared to control cells. Experiments were repeated four times and similar results were obtained.

Caspase activation is a valuable and reliable marker for apoptosis. LNCaP, 22RV1, and DU-145 cells were analyzed for global caspase activation after the treatment with Vitamin K2 (Figure 4). The cells were incubated with 100 *μ*M of VK2 for 2 days. All three cell lines displayed a dramatic increase in apoptosis following treatment (*P* < 0.05), as shown by a high caspase activity after the treatment.

### 3.4. Caspase-3 and Caspase-8 Activity

**Figure 5:**
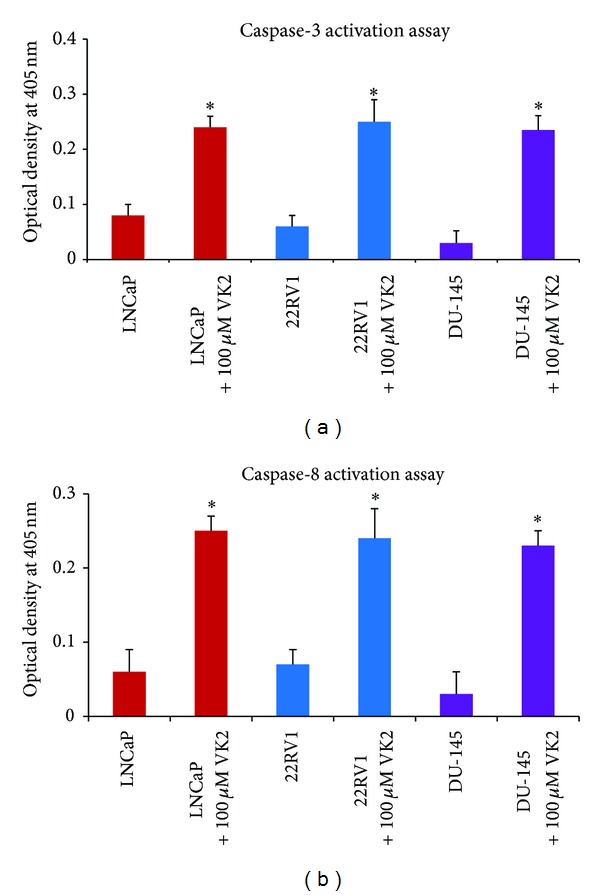
VK2 induced caspase-3 and caspase-8 activity in prostate cancer cells. Detection of caspase-3 and caspase-8 activity in LNCaP, 22RV1 and DU145 prostate cancer cells was measured using a commercially available colorimetric assay kit. Cells were treated with 100 *μ*M of VK2 for 48 hrs. The samples were then analyzed for caspase-3 (Figure 5(a)) and caspase-8 (Figure 5(b)) activity. The results showed that VK2 treatment induced significant activation of caspase-3 and caspase-8 in LNCaP, 22RV1, and DU-145 prostate cancer cells (*P* < 0.05) compared to control treated (0.1% DMSO) prostate cancer cells. This data is representative of one of the three similar experiments.

Caspase-3 and caspase-8 are major death related enzymes that are unregulated when cells undergo apoptosis [22]. To determine whether caspase-3 and caspase-8 enzymes are activated in VK2 treated LNCaP, 22RV1, and DU145 cells, we used a chromogenic based caspase assay. Our results shows that treatment with VK2 induced significant (*P* < 0.05) activation of caspase-3 and caspase-8 in LNCaP, 22RV1, and DU145 cells (Figures 5(a) and 5(b)) compared to control treated cells.

### 3.5. Western Blot Analysis

#### 3.5.1. VK2 Treatment Down Regulates the Expression of Androgen Receptor (AR) in LNCaP Cells

**Figure 6:**
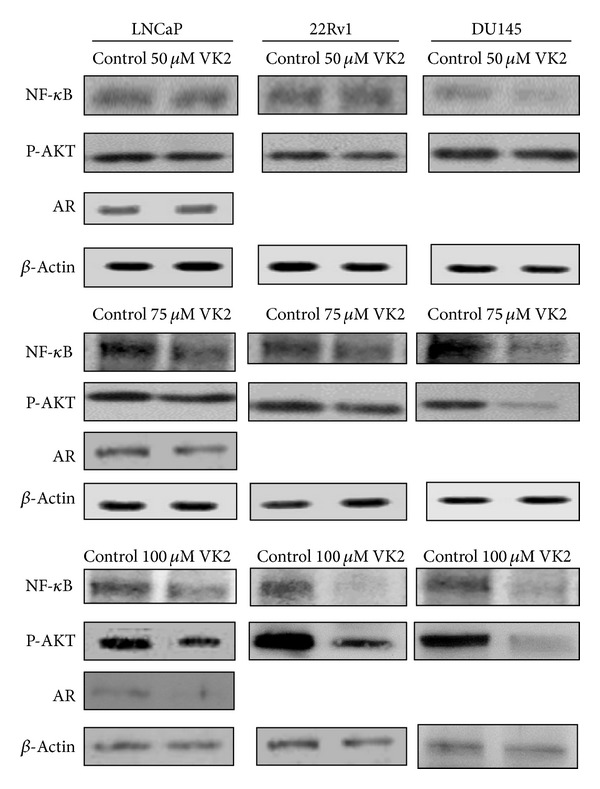
VK2 treatment inhibits transcription factors AR, NF-kB in androgen-dependent and androgen-independent prostate cancer cells. Western blot analysis showed that 100 *μ*M VK2 inhibits the expression of AR, NF-kB, and phospho-AKT in LNCaP, 22RV1 and DU145 prostate cancer cells. LNCaP, 22RV1, and DU145 cells treated with 0.1% DMSO alone served as controls in this experiment.

Our previous results showed PSA expression is substantially reduced by VK2 treatment (Figure 2). AR regulates expression of PSA and is a key regulator of prostate cancer growth. We, therefore, sought to assess the effects of VK2 treatment on AR expression in androgen-dependent prostate cancer cells. Immunoblot analysis of LNCaP cells treated with 50–100 *μ*M VK2 showed reduction in expression of AR compared to control LNCaP cells (Figure 6) suggesting that the decreased PSA in LNCaP cells by VK2 is due to its targeting of AR expression.

#### 3.5.2. VK2 Treatment Downregulates Expression of Phospho-Akt and NF-kB Signaling Molecules in LNCaP, 22RV1, and DU-145 Cells

The serine/threonine protein kinase AKT activation is involved in a variety of cell processes including cell survival and cell invasion in cancer [23]. It is also essential for vascular endothelial growth factor mediated angiogenesis [24]. The phospho AKT pathway has also been implicated as a key pathway in prostate cancer progression [25]. AKT pathway is also implicated in prostate cancer progression and androgen independence [26]. Our results earlier have shown that prostate cancer cell proliferation is significantly reduced in androgen-dependent LNCaP and androgen-independent cell lines, DU145 and 22RV1. We next sought to assess whether VK2 reduces AKT activity. Following treatment of LNCaP, 22RV1 and DU145 cells with 50–100 *μ*M of VK2 for 2 days, immunoblot analysis was carried out. Our results showed a marked reduction in the expression of phospho Akt in all 3 prostate cancer lines in a dose-dependent manner that we assessed. We next sought to determine the effects of VK2 on NF-kB expression. NF-kB (p65) plays a critical role in cell proliferation, antiapoptosis, angiogenesis, and invasion of prostate cancer cells. As our results here show, NF-kB expression is reduced in both androgen-dependent LNCaP cells and androgen-independent 22RV1 and DU145 prostate cancer cells (Figure 6).

#### 3.5.3. Effect of VK2 on the Expression of Inflammatory and Angiogenic Genes in Androgen-Dependent (LNCaP) and Androgen-Independent (22RV1 and DU145) Prostate Cancer Cells

**Figure 7:**
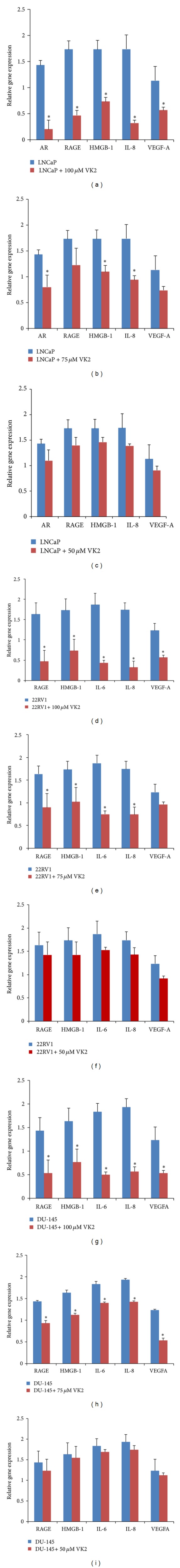
VK2 inhibits the expression of inflammatory cytokine genes: ~1 × 10^6^ LNCaP, DU145, and 22RV1 cells were incubated with 50 *μ*M, 75 *μ*M, and 100 *μ*M Vk2, respectively, for 48 h at 37°C in 5% Co_2_ environment. Untreated cells served as control. Following incubation, RNA was extracted and converted to cDNA. Expressions of HMGB1, RAGE, IL-6, IL-8, VEGF-A, and androgen receptor (AR) genes were then quantified by real time PCR as indicated. Results presented are relative expression levels of genes in the treated samples compared to expression level of the untreated samples. Results show that Vk2 modulates the expression of inflammation related genes by down regulating AR, HMGB1, RAGE, IL-8 and VEGF-A in LNCaP cells ((a)–(c)) compared to control LNCaP cells (*P* < 0.05). In case of 22RV1 ((d)–(f)) and DU-145 (Figures 7(g)–7(i)) prostate cancer cells, VK2 inhibited RAGE, HMGB1, IL-6, IL-8, and VEGF-A expression compared to control cells (*P* < 0.05). Results presented are representative of one of three similar experiments.

Several inflammatory-related genes have been linked with initiation and progression of prostate and other types of cancers [27, 28]. Previous studies showed that inflammatory cytokines IL-6 and IL-8 are associated with prostate cancer [29, 30] and elevated during metastasis [31]. Similarly, overexpression of nuclear binding protein and proinflammatory mediator HMGB1 has been implicated in proliferation and metastasis of many cancer types, including prostate cancer [32]. In a recent study from our laboratory, downregulation of RAGE, a receptor that is implicated in inflammatory process resulted in the induction of apoptosis in prostate cancer cell lines [33]. Hence, we studied the effects of VK2 on inflammatory genes in LNCaP, 22 RV1, and DU-145 cells by quantitative real time RT-PCR. Our results (Figures 7(a)–7(c)) showed that VK2 downregulated the expression of HMGB1, RAGE, IL-8 and VEGF-A in LNCaP cells which are negative for IL-6. On the other hand, VK2 inhibited the expression of IL-6 in 22RV1 and DU-145 cells in addition to downregulating HMGB1, RAGE, IL-8, and VEGF-A in these advanced prostate cancer cells (Figures 7(d)–7(i)).

#### 3.5.4. VK2 Treatment Reduces the Cell Migration of DU145 and 22RV1 Cells *In Vitro*

**Figure 8:**
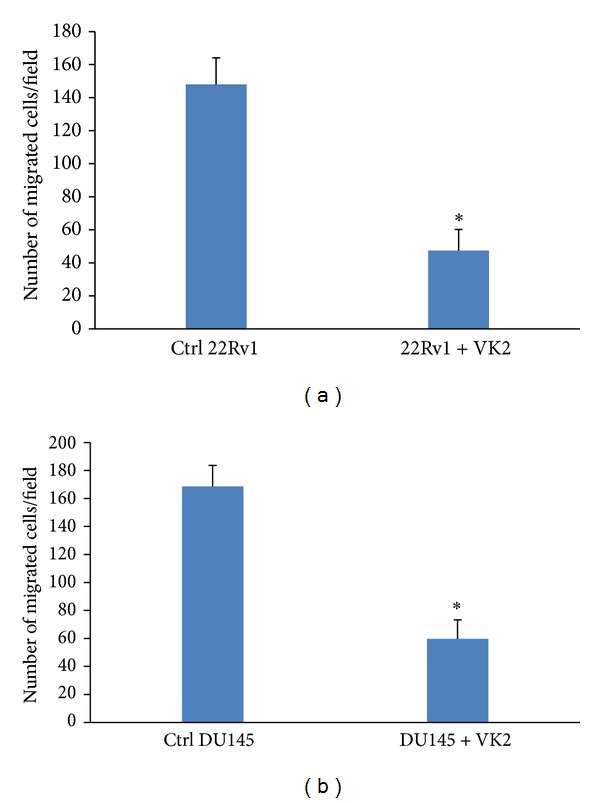
VK2 treatment abrogates migration of prostate cancer cells *in vitro*. Boyden chamber assay shows that control 22RV1 (a) and control DU145 (b) prostate cancer cells have a greater number of migrated cells while 22RV1 and DU145 samples treated with 100 *μ*M of VK2 showed fewer migrated cells in the Transwell chambers. The inhibition of cell migration suggests that VK2 may have anti-migratory properties in prostate cancer. *P < 0.05 compared to control migrated cells. Data shown here is representative of one of three similar results obtained.

One of the first steps in cancer metastasis is cancer cell migration and invasion. Utilizing several lytic enzymes, tumor cells degrade the surrounding extracellular matrix, allowing them to migrate [34]. Treatments aimed at reducing the invasive potential of cancer cells are therefore considered critical. As LNCaP cells are not a good model to study migration, we tested the antimigration potential of VK2 on DU145 and 22RV1 cells using Transwell filters. The results obtained here show that VK2 significantly reduced the number of DU145 and 22RV1 cells capable of migrating the Transwell filters compared to untreated cells (Figure 8). Our results thus suggest that VK2 has antimigratory effects on prostate cancer cells. 

#### 3.5.5. VK2 Treatment Reduces the Angiogenic Potential of DU145 and 22RV1 Cells *In Vivo*

**Figure 9:**
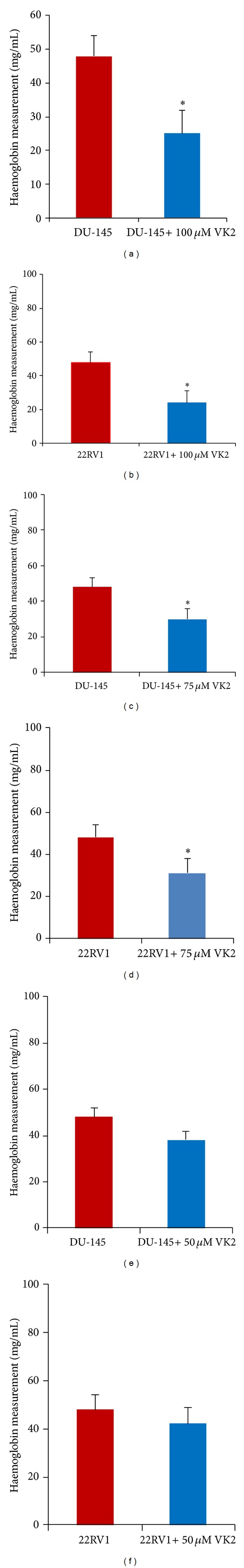
VK2 inhibits hemoglobin levels in matrigel plugs implanted with prostate cancer cells. Angiogenic factors, including inducers and inhibitors, can be added to the matrigel matrix prior to the injection of human DU145 and 22Rv1 cells into nude mice. Matrigel, DU145 or 22RV1 cells and 100 *μ*M of VK2 or the equivalent volume of sterile water were injected into the flanks of nude mice. After a period of 14 days, the gel matrix was recovered and angiogenesis of the recovered matrix gel was quantified using the hemoglobin assay. The hemoglobin level was considerably reduced in the VK2 treated DU145 ((a), (c) and (e)) and 22Rv1 cells ((b), (d), and (f)) (*P* < 0.05) as compared to untreated control. The results are presented as a bar graph. The results shown here are representative of three independent results.

Angiogenesis is one of the hallmarks of cancer progression [35]. Vascular endothelial growth factor, or VEGF-A, is one of the key signals used by oxygen deprived cells to promote neovascularization [36]. Given the reduction in expression of phospho AKT and NF-kB, along with the reduced expression of VEGF-A in DU145 and 22Rv1 prostate cancer cells treated with VK2, we next sought to determine whether treatment with VK2 reduces angiogenesis *in vivo*. To this end, nude mice were implanted subcutaneously with DU145 and 22RV1 cells, along with 50, 75 or 100 *μ*M of VK2 for 2 weeks. Following treatment, the antiangiogenic potential of VK2 was determined using a hemoglobin assay on the matrigel plugs collected from the nude mice. As our results show mice implanted with matrigel containing DU145 and 22RV1 cells treated with VK2 had substantially reduced levels of hemoglobin in the matrigel plug suggesting that VK2 disrupted new blood vessel formation (angiogenesis *in vivo*) that is critical for tumor establishment (Figure 9). 

#### 3.5.6. VK2 Inhibits Tumor Growth of Human Prostate Cancer Cell Xenografts Implanted in Immunodeficient Mice

**Figure 10:**
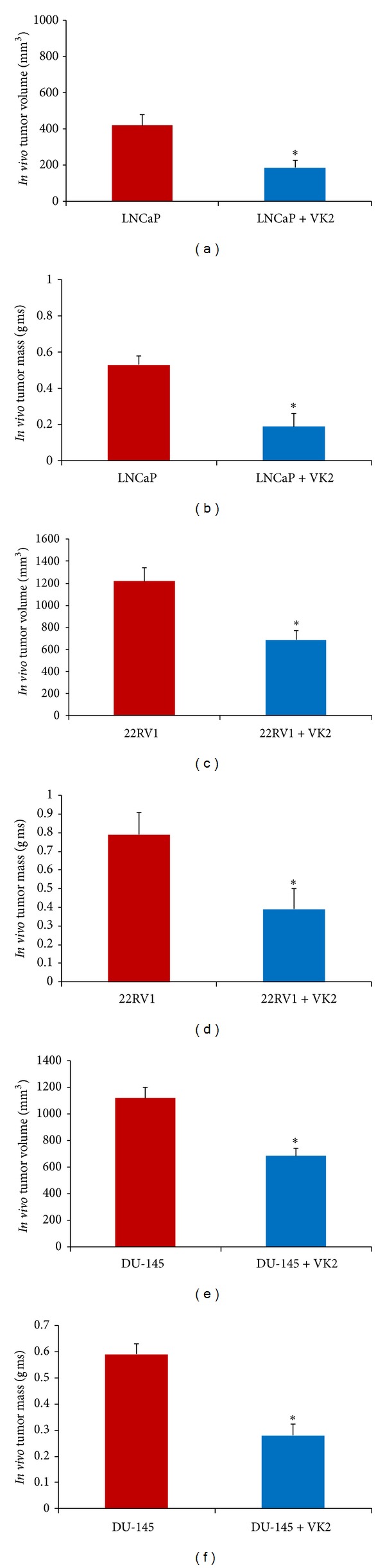
Effects of VK2 on the growth of LNCaP, 22 RV1, and DU145 xenografts in nude mice: VK2 inhibits the growth of LNCaP, 22RV1 and DU145 derived tumor xenografts in nude mice model. Tumor volume (mm^3^) and weights (gms) of the VK2 treated and control untreated tumors of the nude mice were measured at the end of the study. Six independent tumors were collected from the VK2 treated LNCaP, 22RV1, and DU-145 and untreated control nude mice, respectively. Results (a–f) showed that VK2 significantly (*P < 0.05) reduced the tumor weight and tumor volumes of both androgen-dependent and androgen-independent derived prostate cancer cells implanted in nude mice compared to control groups.

We next sought to determine the antitumor effects of VK2 *in vivo *against androgen-dependent and androgen-independent prostate cancer cells. Nude mice were subcutaneously implanted with LNCaP, 22RV1, and DU145 cells. Nude mice in the treatment group were fed water containing VK2 (20 mg/kg/day) for 6 weeks. These results revealed treatment with VK2 significantly reduced LNCaP, 22RV1, and DU145 derived tumor growth *in vivo *(Figure 10). Based upon these results, treatment with VK2 appears to inhibit tumor growth of both androgen-dependent and androgen-independent cells *in vivo*. 

## 4. Discussion

Previous epidemiological studies have linked low dietary intake of VK2 and the development of prostate cancer [16, 17]. Little is known, however, regarding the antitumor potential of VK2 in prostate cancer cell lines. In the present study, we therefore sought to characterize the antitumor potential of VK2 in both androgen-dependent and independent-prostate cancer cells. Apoptosis or programmed cell death is a key process regulating cancer development and progression. Dysregulation of apoptosis represents one of the major hallmarks of cancer [37]. Therapies that cause apoptotic elimination of cancer cells with limited side effects are therefore critical in the treatment of cancer. Our results of this study show that VK2 is able to suppress viability of androgen-dependent and androgen-independent prostate cancer cell lines in a dose dependent manner. We also show here that the reduced proliferation induced by VK2 is the result of caspase-3 and -8 dependent apoptosis. Thus, our study reiterates the apoptotic potential of VK2 against prostate cancer cells as has been previously reported for other cancer types [5–7]. 

The anti-proliferative effect of VK2 was confirmed *in vivo*, where tumor growth was substantially reduced following treatment with VK2 in nude mice subcutaneously injected with LNCaP, 22RV1 and DU145 prostate cancer cells. Our *in vitro* results correlated with *in vivo* results, as androgen-independent 22RV1 and DU145 cells were as sensitive to VK2 treatment as androgen-dependent LNCaP cells. There are several possible explanations for the reduction in cell proliferation noted with VK2 treatment. First, as we show here, treatment with VK2 results in reduced androgen receptor expression. Previous studies have shown that the androgen receptor is essential for cell viability and proliferation of prostate cancer cells [38]. Another possible explanation for the reduction in cell viability noted with VK2 treatment is the inhibition of AKT activation. The AKT activation plays a central role in tumorigenesis, promoting cell growth and survival by suppressing apoptosis [39]. Previous studies suggest that AKT signaling may play a key role in prostate cancer progression, allowing cells to continue to proliferate in an androgen deprived state [40]. Increased AKT phosphorylation has also been associated with higher gleason grade, advanced stage and poor prognosis [41]. Recent studies also show that AR and AKT pathways cross-regulate each other through feedback mechanism and that inhibition of both pathways leads to synergistic reductions of cell proliferation [42]. The therapeutic effects of VK2 may, therefore, also be partly explained by reductions in both androgen receptor expression and AKT activation in LNCaP and 22RV1 derived prostate tumors. Additional studies are needed to further explore the mechanisms by which VK2 may potentially abrogate cross-regulation of AR and AKT pathways. Inhibition of NF-kB represents another possible explanation for the anti-proliferative effect of VK2 that we observed in this study. Previous studies have shown the ability of NF-kB to promote cell growth and proliferation of prostate cancer cells via various mechanisms, including regulation of c-myc, cyclin D1, Bcl-2 and IL-6 [43]. Moreover, suppression of NF-kB has been shown to sensitize cells to apoptosis [44]. Taken together, the anti-proliferative effects of VK2 in androgen dependent and androgen-independent prostate cancer cells is likely, at least in part, due to a reduction in AKT and NF-kB activity. 

Recent studies have also demonstrated that inflammation plays an important role in the initiation and progression of several kinds of cancer including prostate cancer [45, 46]. Mediators of inflammation, such as cytokines, chemokines and various inflammatory cells provide a microenvironment for cancer development. Studies have shown that proinflammatory cytokines such as IL-6, IL-8, HMGB1 and RAGE are elevated in advanced hormone resistant prostate cancers [47–49]. Chemotherapeutic agents which have anti-inflammatory properties should therefore be beneficial in the treatment of hormone resistant prostate cancer. To our knowledge, no previous studies have explored the anti-inflammatory effects of VK2 on cancer cells. We, therefore, set out to explore the anti-inflammatory properties of VK2 on prostate cancer cells. Interestingly, our results showed that VK2 significantly down-regulated proinflammatory mediators HMGB1, IL-6, IL-8, VEGF-A and RAGE. A previous study has demonstrated the significance of HMGB1 expression in prostate cancer [50]. Knockdown of HMGB1 results in apoptotic activation in human prostate cancer cells [49]. HMGB1 is also considered as an angiogenic switch molecule [51] which can interact with NF-kB regulating the expression of VEGF [52]. RAGE, receptor for advanced glycation end products, is another key inflammation transducer that can be activated by HMGB1 in prostate cancer cells [50]. IL-6 is a cytokine involved in immune and hematopoetic activities, implicated in the progression of hormone refractory prostate cancer [53, 54]. In androgen-independent prostate cancer cells, IL-6 acts as an autocrine and paracrine growth factor, with depletion of IL-6 rendering cancer cells sensitive to chemotherapeutic agents. IL-8, another important pro-inflammatory cytokine and growth factor, confers chemotherapeutic resistance by modulating the growth and metastasis of androgen-independent prostate cancer cells [55]. Thus, downregulation of inflammation and angiogenesis related genes by VK2 may be beneficial and attractive for prostate cancer management.

Angiogenesis plays a critical role in tumor progression. Angiogenesis is not only critical for primary tumor growth, but also facilitates tumor invasion and metastasis [56]. Drugs that have successfully targeted anti-angiogenesis, such as antibodies to vascular endothelial growth factor, have improved mortality and progression free survival rates in many types of cancer [57, 58]. However, anti-angiogenic therapy was not successful in prostate cancer so far. Inflammation appears to be a key driving force in the development of angiogenesis [59]. Previous studies have shown the anti-angiogenic potential of VK2 in other cancer cell lines. Yoshiji and colleagues showed that VK2 reduced the angiogenesis potential of hepatocellular cancer cell lines [60]. Given the anti-inflammatory properties of VK2 found earlier in our study, we set out to investigate its effects on angiogenesis and migration. Our results presented in this study show angiogenesis is significantly reduced *in vivo*. Moreover, the motility of prostate cancer cells appears to also be reduced *in vitro*. AKT activity has been shown to be central in regulating tumor angiogenesis. Prior studies have shown that AKT activity is both necessary and sufficient to regulate VEGF, the main inducers of angiogenesis [61]. In this current study, AKT activity is reduced in VK2 treated prostate cancer cells. Taken together, our results suggest that the anti-angiogenic effects of VK2 are partly mediated via reduction in AKT activity and by down regulating inflammation related molecules. Furthermore, a previously conducted prospective EPIC-Heidelberg cohort study [62] suggest that intake of VK2 is associated with a reduced risk of incident and fatal prostate cancer supporting our laboratory findings that VK2 may be a promising natural agent for prostate cancer management.

## 5. Conclusion

**Figure 11:**
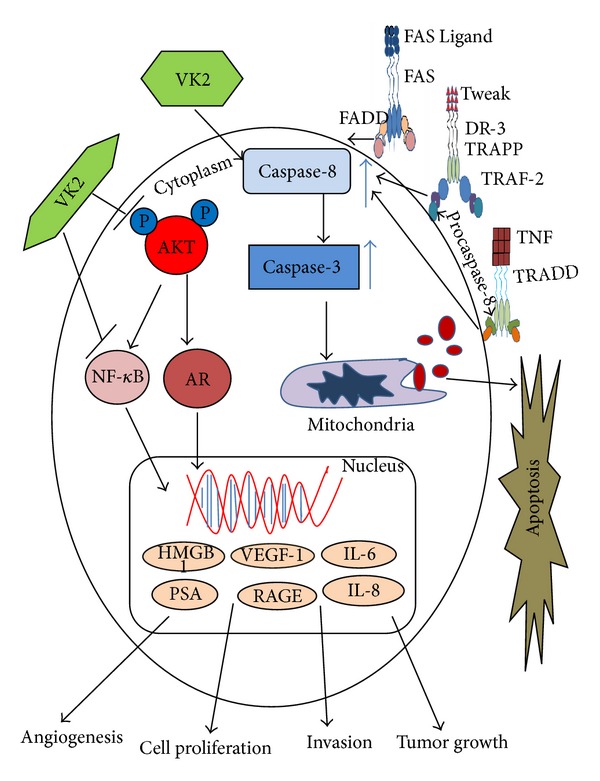
Schematic model depicting the possible mode of VK2 action on prostate cancer cells.

Collectively, the findings in the present study suggest that caspase-3 induction, downregulation of phosphorylated AKT, inhibition of NF-kB, and reduction of androgen receptor expression may represent the molecular mechanisms by which VK2 reduces cell proliferation, induces apoptosis, and reduces the angiogenic potential of prostate cancer cells as outlined in Figure 11. Based upon the previous epidemiological studies of vitamin k2 in prostate cancer [16, 17, 62] and the results presented here, further investigations are warranted to elucidate the therapeutic potential of VK2 against androgen-dependent and androgen-independent prostate cancer.
